# Understanding the association between caregiver sex and HIV infection among orphans and vulnerable children in Tanzania: learning from the USAID Kizazi Kipya project

**DOI:** 10.1186/s12913-020-05102-y

**Published:** 2020-04-03

**Authors:** Amon Exavery, John Charles, Erica Kuhlik, Asheri Barankena, Alison Koler, Levina Kikoyo, Elizabeth Jere

**Affiliations:** 1 Pact, P.O. Box 6348, Dar es Salaam, Tanzania; 2 Pact, Inc., 1828 L Street NW, Suite 300, Washington, DC, 20036 USA

**Keywords:** Orphans, Children, OVC, HIV/AIDS, Caregiver, Sex, At risk populations, HIV testing services, USAID Kizazi Kipya, Tanzania

## Abstract

**Background:**

Tanzania has met only 50.1% of the 90% target for diagnosing HIV in children. The country’s pediatric case finding strategy uses global best practices of index testing, provider-initiated counselling and testing, and targeted community testing of at-risk populations to find about 50,000 children living with HIV (CLHIV) who are undiagnosed. However, context-specific strategies are necessary to find the hidden children to meet the full 90% target. This study assesses whether sex of the caregiver is associated with HIV status of orphans and vulnerable children (OVC) as a valuable strategy for enhanced pediatric case findings.

**Methods:**

Data originate from the community-based, United States Agency for International Development (USAID)-funded Kizazi Kipya Project, which works towards increasing OVC’s and their caregivers’ uptake of HIV/AIDS and other health and social services in Tanzania. Included in this study are 39,578 OVC ages 0–19 years who the project enrolled during January through March 2017 in 18 regions of Tanzania and who voluntarily reported their HIV status. Data analysis involved multi-level logistic regression, with OVC HIV status as the outcome of interest and caregiver’s sex as the main independent variable.

**Results:**

Three-quarters (74.3%) of the OVC included in the study had female caregivers, and their overall HIV prevalence was 7.1%. The prevalence was significantly higher (*p* < 0.001) among OVC with male caregivers (7.8%) than among OVC with female caregivers (6.8%), and indeed, multivariate analysis showed that OVC with male caregivers were significantly 40% more likely to be HIV-positive than those with female caregivers (OR = 1.40, 95% CI 1.08–1.83). This effect was the strongest among 0–4 year-olds (OR = 4.02, 95% CI 1.61–10.03), declined to 1.72 among 5–9 year-olds (OR = 1.72, 95% CI 1.02–2.93), and lost significance for children over age 9 years. Other significant factors included OVC age and nutritional status; caregiver HIV status and marital status; household health insurance status, and family size; and rural versus urban residence.

**Conclusions:**

OVC in Tanzania with male caregivers have a 40% higher likelihood of being HIV-positive than those with female caregivers. HIV risk assessment activities should target OVC with male caregivers, as well as OVC who have malnutrition, HIV-positive caregivers, or caregivers who do not disclose their HIV status to community volunteers. Further, younger HIV-positive OVC are more likely to live in rural areas, while older HIV-positive OVC are more likely to live in urban areas. These factors should be integrated in HIV risk assessment algorithms to enhance HIV testing yields and pediatric case-finding in the OVC population in Tanzania.

## Background

The human immunodeficiency virus (HIV) that causes acquired immunodeficiency syndrome (AIDS) [1, 2] remains a global threat [3, 4]. The UNAIDS estimates that there are 36.9 million people living with HIV/AIDS (PLHIV) worldwide, a majority of whom are in developing countries, 18.2 million are women aged 15+ years, and 1.8 million are children under 15 years of age [5]. In 2016, UNAIDS estimated Tanzania’s overall HIV prevalence among adults at 4.7% [6], and in 2018, UNICEF estimated an HIV prevalence of 0.4% among children [7] under age 15 years in Tanzania [8]. This prevalence was also reported by the 2016–2017 Tanzania HIV Impact Survey [9]. Further estimates by the UNAIDS show that in 2018, there were 92,000 (72,000–110,000) children living with HIV, 8600 (6500–13,000) children newly infected with HIV, and 5400 (3200–8900) child deaths due to AIDS in Tanzania [10].

### Methods of and factors leading to transmission of HIV in children

Vertical transmission, commonly known as mother-to-child transmission of HIV (MTCT) is the predominant mode through which children acquire HIV [11, 12]. Other routes include blood transfusions and the use of contaminated sharp objects [13]. In communities affected by AIDS, children who have lost parents and family members become more vulnerable to HIV infection from the lack of caregivers, lack of access to school and inability to stand for their rights; in these cases, children can be infected through sexual abuse or rape [14]. Prevention of mother-to-child transmission of HIV (PMTCT) services during pregnancy, delivery and breastfeeding can stop MTCT [15], but relying primarily on this approach does not address the challenge of maternal seroconversion during late pregnancy and breastfeeding [16, 17] and creates coverage gaps [18].

In the areas heavily affected by HIV/AIDS, such as sub–Saharan Africa, the association of orphanhood and AIDS is well established [19–21]. Orphanhood, which occurs when a child under 18 years of age loses one or both parents [11], increases HIV risk in children. For example, orphans are two to three times more likely than their non-orphaned counterparts to have acquired HIV by the time they reach adolescence [22, 23]. In 2016, Tanzania had 2.6 million orphans from all causes, 810,000 of whom were orphaned by AIDS [24]. The magnitude of orphanhood in the country increases with age, from as low as 1% in children under age 2 years to as high as 18% in children aged 15–17 years [25]. Orphanhood also varies by geographical location, with the highest rates in Iringa (13%), Ruvuma (12%), and Mara (12%) regions [25]. However, widespread access to antiretroviral therapy (ART) has led to increased survival among HIV-positive caregivers, reducing cases of children orphaned due to HIV [26].

In this context, pediatric HIV testing, care and treatment must receive the same attention and resources as PMTCT [27]. Pediatric HIV care has lagged as a result of weak and fragmented systems for pediatric case finding. Consequently, many children die of HIV, often undiagnosed [27].

### The 90–90-90 targets

In 2014, at the 20th International AIDS Conference in Melbourne, Australia, UNAIDS launched the 90–90-90 targets for HIV/AIDS programming, which state that by 2020, 90% of all PLHIV will know their status, 90% of people diagnosed with HIV will be on ART, and 90% of people on ART will achieve viral suppression [28]. Essentially, early identification, prompt and sustained treatment, and viral suppression can prevent the transmission of HIV, thus reducing HIV incidence at a population level [29].

While the first 90 is the parent of the subsequent 90s in the cascade, its performance gap is largest [30], with only 75% of people worldwide knowing their status in 2017 [31]. Tanzania’s progress towards achieving the 90–90-90 targets mirrors the global trend at 61–94-87 among adults [9]. Progress in the pediatric population also lags behind, with only 50.1% of Tanzanian children living with HIV (CLHIV) diagnosed [9]. Therefore, a priority for the country is identifying and linking to care all individuals, and particularly children, who could be infected but are unaware of their HIV status [9].

### Gaps in testing coverage and efficacy for orphans and vulnerable children (OVC)

Most HIV-positive children are diagnosed late and at an advanced stage of disease progression [32]. Further, evidence shows that without ART, 53% of CLHIV die before their second birthday [33]. Most of these children are born to women who do not access or who only partially access PMTCT services [34]. A substantial proportion of these deaths could have been prevented if the children were identified, diagnosed, and initiated on treatment. But, pediatric HIV case-finding remains challenging [34, 35], particularly access to HIV testing services (HTS) for children.

Factors associated with HIV status in children have been identified in previous studies and can demonstrate heightened risk for acquiring HIV, thus an increased need to focus HTS for specific groups. For example, maternal CD4 count during pregnancy, mixed feeding, and being hospitalized since birth were noted among children born to mothers in PMTCT programs in Zimbabwe [36]. A study among HIV exposed children in Uganda observed that infants who did not receive ART prophylaxis at birth and children delivered outside the health facility were more likely to be HIV-positive than their counterparts [37]. Significant association between malnutrition and HIV status in children has been observed in many countries, including Burkina Faso, Ghana, Rwanda, and India [38–42]. Other studies have noted a higher likelihood of HIV infection in children living with HIV-positive caregivers [43]. However, the literature lacks adequate evidence of HIV prevalence among OVC and corresponding risk factors. The association between caregiver sex and OVC HIV status is missing. Given the links between orphanhood and HIV, knowing how caregiver sex and other individual and household characteristics associate with OVC HIV status is crucial for informing pediatric case-finding strategies to ultimately close the pediatric gap in the first 90.

Tanzania offers index testing services to children if the biological mother is HIV-positive or if the father is HIV-positive and reports that the child’s mother is HIV-positive, deceased, or of unknown status and/or that a biological sibling under age 15 years is HIV-positive [44]. However, if the father comes for HTS and tests HIV-negative, nothing else is enquired under the current index testing algorithm. While children with female caregivers are further covered by HTS under the current index testing algorithm, children under the direct care of these fathers will be missed. The problem is compounded by a lower predilection for health-seeking, including HIV testing, among men than among women [45, 46]. This highlights a need for a critical analysis of whether caregiver sex is associated with HIV infection in children, especially OVC. This will inform further targeted efforts toward diagnosis targets and to further improvement in pediatric case-finding modalities for universal coverage of HTS for children, including orphans and vulnerable ones.

## Methods

### Data source

Data are from Pact’s community-based, USAID-funded Kizazi Kipya Project in Tanzania (2016–2021). The project aims to increase the uptake of HIV and other health and social services by OVC and their household members. Community Case Workers (CCWs) collected the data from caregivers’ self-reports during beneficiary enrollment using the project’s Screening and Enrollment, and Family and Child Asset Assessment (FCAA) tools. Beneficiaries are enrolled into the project if their household meets one or more of the 14 enrollment criteria that cover household vulnerabilities related to HIV: household is headed by a child (under age 18 years), household is headed by an elderly caregiver (age 60 years or older), household cares for at least one single or double orphan, caregiver is chronically ill and unable to meet his/her children’s basic needs, caregiver is a drug user, caregiver or an adolescent age 10–19 years in the household is a sex worker, at least one adolescent girl age 10–19 years in the household is sexually active, adolescent girl age 10–19 years in the household is pregnant or has a child of her own, at least one household member is HIV-positive, at least one child in the household has tuberculosis, at least one child in the household is severely malnourished, at least one child in the household has been or is being abused or at risk of abuse, at least one child in the household is living and/or working on the streets, and at least one child in the household is working in mines. These criteria are equally applied for all implementation areas and age groups.

### Study area

Data for this study originated from 18 regions of Tanzania where the USAID Kizazi Kipya project had implemented enrollment activities in 2017: Dar es Salaam, Dodoma, Geita, Iringa, Kagera, Katavi, Mbeya, Mjini Magharibi, Morogoro, Mtwara, Mwanza, Njombe, Pwani, Rukwa, Ruvuma, Singida, Tabora, and Tanga. Of these regions, Mjini Magharibi has very low adult HIV prevalence (0.6%), while Njombe has the highest in the country (11.4%) according to the recent Tanzania HIV Impact Survey [9]. A total of 67 district councils (48 rural and 19 urban) considered high HIV burden from the 18 regions were included in this study.

### Study population

The study population encompassed 39,578 OVC who were enrolled in the USAID Kizazi Kipya Project from January to March 2017, and had complete information on their HIV status and their caregivers’ characteristics. In the context of the U.S. President’s Emergency Plan for AIDS Relief (PEPFAR), an OVC is a child, ages 0–17 years, who is either orphaned (i.e. lost one or both parents to HIV/AIDS) or made more vulnerable because of HIV/AIDS [47]. For programming purposes, the USAID Kizazi Kipya Project extends the OVC age to 19 to include all adolescents [48]. Therefore, OVC included in this study were aged 0–19 years. The majority of the OVC were under age 15 years (*n* = 27,935, 70.6%).

USAID Kizazi Kipya defines a *caregiver* as a guardian with the greatest responsibility for the daily care and rearing of one or more OVC in a single household. A caregiver is not necessarily a biological parent. Only one caregiver per OVC was included in this study: the person identified as having primary responsibility for caring for the child, i.e., the *primary caregiver*. The youngest caregiver in this study was aged 18 years. References to caregiver in this manuscript denote each child’s singular, primary caregiver.

### Study design

The study design constituted a cross-sectional secondary analysis of the existing FCAA data, as described above. The data were collected once during beneficiary screening and enrollment.

### Variables

*OVC HIV status as reported by the caregiver* was the outcome or dependent variable and was measured through the two categories of *negative* and *positive*. For computational purposes, the variable was organized as follows:

$$ \mathrm{OVC}\ \mathrm{HIV}\ \mathrm{status}=\Big\{{\displaystyle \begin{array}{c}0,\mathrm{if}\ \mathrm{the}\ \mathrm{OVC}\ \mathrm{was}\ \mathrm{reported}\ \mathrm{HIV}-\mathrm{NEGATIVE}\\ {}1,\mathrm{if}\ \mathrm{the}\ \mathrm{OVC}\ \mathrm{was}\ \mathrm{reported}\ \mathrm{HIV}-\mathrm{POSITIVE}\end{array}} $$

The main independent variable for this study was *sex of the caregiver*, measured through the two categories of *male* and *female*. Other independent variables included OVC sex, OVC age (in years), OVC nutritional status, caregiver age (in years), HIV status of the caregiver, education of the caregiver, family size, whether some or all the family members are covered by health insurance, whether the caregiver is physically or mentally disabled, household wealth quintile, and type of residence (rural or urban). Rural residence included all those living in district councils, whereas those living in township, municipal or city councils were considered as urban residents.

Family size (i.e., number of people living in the same household) was divided into three categories: households with 2–3 members, households with 4–13 members, and households with 14 or more members. This was based on an explorative analysis of OVC HIV prevalence by family size as a discrete variable. Families with similar prevalence were grouped together, thus the categories. The smallest household had two occupants – the OVC and his/her caregiver.

Nutritional status was assessed using mid-upper arm circumference (MUAC) measuring tapes. MUAC is recommended for community-based screening of acute malnutrition [49]. Interpretation of the readings was guided by the standard definitions of the colors, whereby the person being assessed is nourished if the reading falls in the tape’s green zone, the person being assessed is moderately undernourished if the reading falls in the yellow zone, and the person being assessed is severely undernourished if the reading falls in the red zone [50].

Wealth quintile was constructed using principal component analysis (PCA) of household assets to determine household socio-economic status [51]. Five wealth quintiles were formed, ranging from the lowest quintile (Q1) for the poorest households, to the highest quintile (Q5) for the most well-off households. Family-owned assets included in the PCA were dwelling materials (brick, concrete, cement, aluminium, other), livestock (chicken, goats, cows, other), transportation assets (bicycle, motorcycle/moped, tractor, motor vehicle, other), and productive assets (sewing machine, television, couch/sofa, cooking gas, hair dryer, radio, refrigerator, blender, oven, other).

### Data analysis

Data analysis was conducted using Stata version 14.0 statistical software. Exploratory analysis was conducted through one-way tabulations to obtain distributional features of the respondents in each variable. Cross-tabulation of OVC HIV status by each of the independent variables was conducted to assess the variability of OVC HIV prevalence by levels of each of the independent variables. The Chi-Square (χ^2^) test was used to assess the degree of association between OVC HIV status and each of the independent variables.

Multivariate analysis was conducted using a random-effects logistic regression model due to the hierarchical or clustered structure of the data [52]. The usual assumption of independence of the observations did not hold because two or more OVC who have the same caregiver, or who reside in the same household may be correlated. Thus, a multilevel model, which recognizes these data hierarchies and allows for residual components at each level in the hierarchy, was used [53]. This choice was based on the assumptions that OVC from the same household and caregiver are dependent in their behavioral, physical, or mental characteristics because they share the same social, health, and economic resources, as well as risks available at the household level. This is likely to exert a related influence in their social life and health outcomes.

Five multivariate models were constructed. The first model encompassed the entire study population of 39,578 OVC ages 0–19 years. The remaining models broke down the study population by age group: the second model was for 5217 OVC in the age group 0–4 years, the third model was for 10,457 OVC in the age group 5–9 years, the fourth model was for 12,261 OVC in the age group 10–14 years, and the fifth model was for 11,643 OVC in the age group 15–19 years. The stratification of the multivariate analysis by OVC age offered a deeper examination, interpretation and comparisons of the patterns and concentration of the association between caregiver sex and OVC HIV status across different bands of the OVC age.

All statistical inferences were made at the conventional significance level of 5% (α = 0.05), whereby any association corresponding with a *p*-value less than 0.05 was considered statistically significant.

### Limitations

Some key variables, such as whether the caregiver was the child’s biological parent, were not available in the data. Recall bias was possible during data collection because all information (except for nutritional status, which was measured) was self-reported, though findings suggest that the effect may be minimal because results are comparable with existing biomedical and clinical studies. Since this study was cross-sectional in design, temporality cannot be established, which precludes drawing causal inferences from these findings.

## Results

### Profile of OVC

Table 1 shows frequency distribution of the OVC analyzed. The study included 39,578 OVC ages 0–19 years (mean = 10.9, and standard deviation [SD] = 5.0). The OVC age groups of 0–4, 5–9, 10–14, and 15–19 years constituted 13.2, 26.4, 31.0, and 29.4% of the total sample respectively. There was no distributional difference in sex of the OVC; about half (51.3%) of the OVC study population was female. With respect to OVC nutritional status, majority (58.5%) were nourished. In terms of HIV status, 7.1% (*n* = 2802) of the OVC were reported HIV-positive.

**Table 1:** Profile of OVC

Variable	Number of OVC (n)	Percent of OVC (%)
**OVERALL**	39,578	100.0
**OVC HIV status**		
**Negative**	36,776	92.9
**Positive**	2802	7.1
**Caregiver sex**		
Female	29,401	74.3
Male	10,177	25.7
**Caregiver age group (in years)**		
18—29	2280	5.8
30—39	9283	23.5
40—49	11,571	29.2
50—59	6847	17.3
60+	9597	24.2
Mean = 48.7, SD = 14.1, Median = 46, Min = 18, Max = 110	–	–
**Caregiver marital status**		
Married or living together	17,833	45.1
Divorced or separated	5061	12.8
Never married (single)	2584	6.5
Widowed	14,100	35.6
**Family size**		
2–3 people	9440	23.9
4–13 people	29,944	75.7
≥ 14 people	194	0.5
**Caregiver mentally or physically disabled?**		
No	37,967	95.9
Yes	1611	4.1
**OVC sex**		
Female	20,304	51.3
Male	19,274	48.7
**OVC age group (in years)**		
0—4	5217	13.2
5—9	10,457	26.4
10—14	12,261	31.0
15—19	11,643	29.4
Mean = 10.9, SD = 5.0. Median = 11, Min = 0, Max = 19	–	–
**Caregiver HIV status**		
Negative	21,818	55.1
Positive	10,398	26.3
Undisclosed	7362	18.6
**Wealth Quintile**		
Lowest (Q1)	10,842	27.4
Second	5348	13.5
Middle	6794	17.2
Fourth	7575	19.1
Highest (Q5)	9019	22.8
**Caregiver education**		
Never attended	7676	19.4
Primary	30,569	77.2
Secondary or higher	1333	3.4
**OVC nutritional status (MUAC)**		
Green (healthy)	23,156	58.5
Yellow (moderately malnourished)	1416	3.4
Red (severely malnourished)	144	0.4
Unknown	14,862	37.6
**Household member(s) has health insurance card**		
Yes	5813	14.7
No	33,765	85.3
**Place of residence**		
Rural	21,585	54.5
Urban	17,993	45.5

Three-quarters of the OVC (74.3%) were living with a primary caregiver who was female. These OVC had caregivers with mean age of 48.7 [SD = 14.1] years. Nearly half (45.1%) of the OVC were living with married caregivers, and 35.6% were living with widowed caregivers. More than three–quarters of the OVC (77.2%) had caregivers with primary education. Just over half of the OVC (54.5%) lived in rural areas. Regarding family size, the majority (75.7%) of OVC were living in families with 4–13 people. Slightly more than a quarter (26.3%) of the OVC were living with HIV-positive caregivers.

### OVC HIV status by background characteristics

**Fig. 1:**
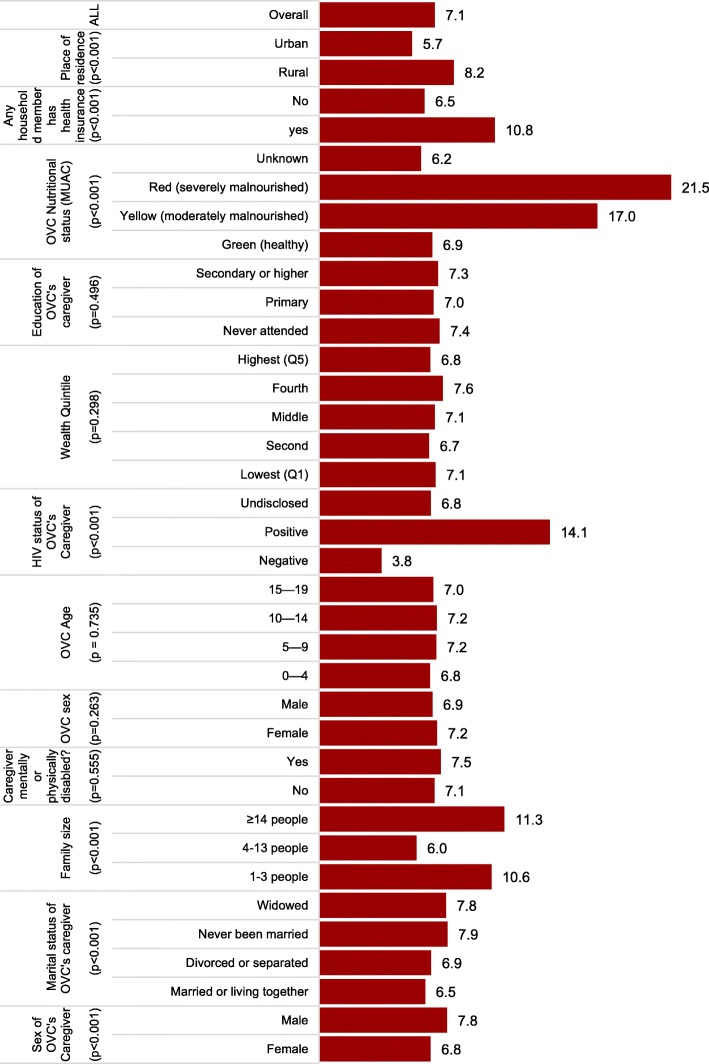
Percentage of HIV-positive OVC in Tanzania, by background characteristics, 2017 (*n* = 39,578)

OVC HIV prevalence by each of the independent variables is presented in Fig. 1.

Overall 7.1% (*n* = 2802) of OVC were reported HIV-positive. This proportion varied significantly by levels of several independent variables. With respect to caregiver sex, OVC HIV prevalence was significantly higher (*p* < 0.001) among OVC with male caregivers than those with female caregivers (7.8 and 6.8%, respectively). HIV prevalence was lowest (3.8%) among OVC living with HIV-negative caregivers and highest (14.1%) among OVC living with HIV-positive caregivers (*p* < 0.001). OVC HIV status varied significantly by OVC nutritional status (*p* < 0.001), whereby HIV prevalence among OVC who were severely malnourished, moderately malnourished, and nourished was 21.5, 17.0 and 6.9% respectively. Caregiver’s marital status was also associated with OVC HIV status (*p* < 0.001), with OVC HIV prevalence being lowest (6.5%) among OVC with caregivers who were married or living together and highest (7.9%) among OVC living with caregivers who were never married.

**Fig. 2:**
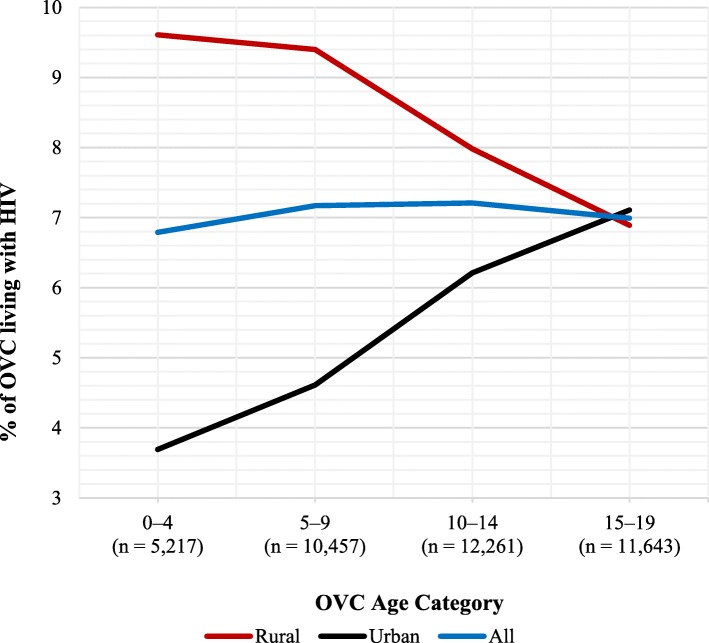
Rural-urban differentials in HIV prevalence among OVC in Tanzania, by age group, 2017 (*n* = 39,578)

HIV prevalence also varied by family size; it was highest among OVC living in households with 14 or more, and 1–3 household members (11.3 and 10.6% respectively) and lowest (6.0%) among OVC living in households with 4–13 members (*p* < 0.001). With respect to location, HIV prevalence was significantly higher among OVC living in rural areas than in urban areas (8.2 and 5.7%, respectively) (*p* < 0.001). As detailed in Fig. 2, HIV prevalence among OVC living in rural areas was highest in the youngest age group (0–4 years) and continued declining as OVC age advanced. After age 15 years, the OVC HIV prevalence in urban areas surpassed that in the rural areas. The proportion of OVC living with HIV varied by health insurance ownership, with the prevalence highest (10.8%) among OVC living in households with health insurance and lowest (6.5%) in households without health insurance.

HIV prevalence among OVC was not significantly associated with the caregiver’s education level (*p* = 0.496), household wealth quintile (*p* = 0.298), OVC sex (*p* = 0.263), or caregiver disability status (*p* = 0.555).

### Results from the multivariate analysis

Adjusted odds ratios (OR) and their corresponding 95% confidence intervals (CIs) for the association between caregiver’s sex and OVC HIV status are presented in Table 2.

**Table 2:** Multivariate random-effects logistic regression models of the association between caregiver sex and OVC HIV infection in Tanzania, 2017 (*n* = 39,578)

Variable	ALL OVC (***n*** = 39,578)		OVC Age group							
			Age 0–4 years (***n*** = 5,217)		Age 5–9 years (***n*** = 10,457)		Age 10–14 years (***n*** = 12,261)		Age 15–19 years (***n*** = 11,643)	
	Odds Ratio (OR)	95% Confidence Interval (CI)	Odds Ratio (OR)	95% Confidence Interval (CI)	Odds Ratio (OR)	95% Confidence Interval (CI)	Odds Ratio (OR)	95% Confidence Interval (CI)	Odds Ratio (OR)	95% Confidence Interval (CI)
**OVC's caregiver sex**										
Female	1.00	—	1.00	—	1.00	—	1.00	—	1.00	—
Male	**1.40	1.08—1.83	**4.02	1.61—10.03	**1.72	1.02—2.93	1.32	0.83—2.10	1.07	0.68—1.68
**Caregiver marital status**										
Married or living together	1.00	—	1.00	—	1.00	—	1.00	—	1.00	—
Divorced or separated	1.05	0.74—1.50	2.22	0.78—6.31	1.52	0.76—3.04	1.09	0.59—2.02	**0.51	0.26—0.99
Never been married	**1.63	1.04—2.57	2.54	0.63—10.27	2.03	0.82—5.01	1.87	0.83—4.20	1.56	0.71—3.44
Widowed	1.19	0.91—1.54	**2.58	1.07—6.26	1.31	0.77—2.22	1.32	0.84—2.10	0.91	0.59—1.42
**Family size**	***0.82	0.78—0.87	***0.66	0.54—0.81	**0.84	0.76—0.93	***0.76	0.69—0.84	***0.74	0.67—0.82
**OVC sex**										
Female	1.00	—	1.00	—	1.00	—	1.00	—	1.00	—
Male	0.92	0.79—1.08	0.71	0.39--1.29	0.87	0.60—1.26	0.88	0.63—1.22	0.84	0.60—1.16
**OVC age group (in years)**										
0—4	1.00	—	—	—	—	—	—	—	—	—
5—9	**1.35	1.04—1.75	—	—	—	—	—	—	—	—
10—14	**1.44	1.11—1.86	—	—	—	—	—	—	—	—
15—19	**1.37	1.05—1.79	—	—	—	—	—	—	—	—
**OVC Nutritional status (MUAC)**										
Green (healthy)	1.00	—	1.00	—	1.00	—	1.00	—	1.00	—
Yellow (moderately malnourished)	***11.03	7.40—16.44	***13.24	3.43—51.17	***11.23	4.44—28.42	***32.57	11.67—90.88	***29.52	10.02—87.02
Red (severely malnourished)	***12.71	4.36—37.06	***147.25	6.91—3137.2	0.90	0.04—22.45	**11.69	1.14—119.39	***153.22	13.39—1753.2
Unknown	1.23	0.99—1.52	1.57	0.72—3.41	0.97	0.60—1.57	1.20	0.79—1.80	1.16	0.78—1.71
**OVC's caregiver HIV status**										
Negative	1.00	—	1.00	—	1.00	—	1.00	—	1.00	—
Positive	***29.27	21.04—40.72	***51.65	11.07—240.9	***67.69	25.60—179.0	***33.74	15.24—74.67	***13.94	7.34—26.45
Undisclosed	***3.29	2.43—4.47	2.50	0.90—7.01	***4.10	2.07—8.14	***5.25	2.87—9.57	***3.47	2.03—5.95
**Wealth Quintile**										
Lowest (Q1)	1.00	—	1.00	—	1.00	—	1.00	—	1.00	—
Second	0.84	0.58—1.22	0.61	0.20—1.83	1.23	0.61—2.49	0.63	0.33—1.23	0.74	0.38—1.44
Middle	1.09	0.78—1.52	1.94	0.73—5.14	1.10	0.56—2.13	1.13	0.63—2.01	0.90	0.50—1.61
Fourth	1.29	0.93—1.78	1.10	0.41—2.98	1.68	0.87—3.22	1.41	0.80—2.46	1.04	0.59—1.83
Highest (Q5)	**1.42	1.03—1.96	1.05	0.39—2.88	1.37	0.71—2.64	1.25	0.71—2.18	1.65	0.96—2.82
**Education of the OVC's caregiver**										
Never attended	1.00	—	1.00	—	1.00	—	1.00	—	1.00	—
Primary	0.78	0.59—1.03	0.71	0.31—1.66	0.59	0.33—1.03	0.86	0.53—1.40	0.88	0.54—1.43
Secondary or higher	0.86	0.46—1.60	0.57	0.08—4.17	0.43	0.11—1.63	0.72	0.22—2.35	1.44	0.51—4.01
**Household member(s) has health insurance card**										
Yes	1.00	—	1.00	—	1.00	—	1.00	—	1.00	—
No	***0.34	0.25—0.45	**0.28	0.11—0.73	***0.28	0.15—0.52	***0.38	0.22—0.65	***0.29	0.17—0.50
**Place of residence**										
Rural	1.00	—	1.00	—	1.00	—	1.00	—	1.00	—
Urban	**0.71	0.55—0.91	***0.16	0.06—0.43	***0.38	0.22—0.65	0.81	0.53—1.26	**1.59	1.04—2.44
Constant	0.0017	0.0009-0.0035	0.0009	0.0001-0.0172	0.0007	0.0001-0.0045	0.001	0.0002-0.0053	0.0028	0.0007-0.0113
rho (intraclass correlation coefficient)	0.85	0.83-0.87	0.89	0.79-0.95	0.88	0.82-0.92	0.87	0.81-0.91	0.86	0.80-0.90
Model statistics	Number of OVC = 39,578		Number of OVC = 5,217		Number of OVC = 10,457		Number of OVC = 12,261		Number of OVC = 11,643	
	Number of households = 20,082		Number of households = 4,477		Number of households = 8,165		Number of households = 9,586		Number of households = 9,011	
	OVC per household: min = 1, avg = 2, max = 9		OVC per household: min = 1, avg = 1.2, max = 6		OVC per household: min = 1, avg = 1.3, max = 7		OVC per household: min = 1, avg = 1.3, max = 7		OVC per household: min = 1, avg = 1.3, max = 6	

Statistical significance: ***p < 0.001, **p < 0.05

The overall results show that OVC with male caregivers were significantly and independently 40% more likely to be HIV-positive than OVC with female caregivers (OR = 1.40, 95% CI 1.08–1.83). This effect was four times stronger for OVC ages 0–4 years (OR = 4.02, 95% CI 1.61–10.03) and almost twice as high for OVC ages 5–9 years (OR = 1.72, 95% CI 1.02–2.93). These effects were adjusted for OVC sex, OVC nutritional status, caregiver marital status, caregiver education, place of residence, caregiver HIV status, family size, wealth quintile, and whether some or all household members have health insurance. The effects also were adjusted for the correlation between OVC who reside in the same household (co-residence) or OVC with the same caregiver. The intra-class correlation coefficient (ICC) was 0.85, indicating that the household-level random effect accounted for 85% of the total residual variance. This meant that OVC were more likely to be HIV-positive if they were residing in the same household as other OVC.

There were other factors with significant association with OVC HIV status. The largest effect was exerted by caregiver HIV status, whereby OVC with HIV-positive caregivers were 29.27 times more likely to be HIV-positive than OVC with HIV-negative caregivers (OR = 29.27, 95% CI 21.04–40.72). These effects remained statistically significant with their direction unchanged across all OVC age groups. Similarly, both OVC who were moderately undernourished (OR = 11.03, 95% CI 7.40–16.44) and severely undernourished (OR = 12.71, 95% CI 4.36–37.06) were more likely to be HIV-positive than those who were nourished.

OVC in each of the age groups of 5–9 years (OR = 1.35, 95% CI 1.04–1.75), 10–14 years (OR = 1.44, 95% CI 1.11–1.86), and 15–19 years (OR = 1.37, 95% CI 1.05–1.79) were more likely to be reported HIV-positive than OVC in the youngest age group (0–4 years). A one-person increase in family size resulted in 18% less likelihood of an OVC being reported HIV-positive (OR = 0.82, 95% CI 0.78–0.87). OVC living in households without health insurance were 66% less likely than those living in households with health insurance to be reported HIV-positive (OR = 0.34, 95% CI 0.25–0.45); this effect remained statistically significant and unaltered in direction across all the OVC age groups.

Overall, OVC residing in urban areas were 29% less likely to be reported HIV-positive than their rural counterparts (OR = 0.71, 95% CI 0.55–0.91). The magnitude of this effect was the strongest among the youngest OVC (0–4 years), whose likelihood of being HIV-positive was 84% less in urban than in rural areas (OR = 0.16, 95% CI 0.06–0.43). The direction of the effect changed among 15–19 year-old OVC residing in urban areas, who were 59% more likely to be HIV-positive than their rural counterparts of the same age (OR = 1.59, 95% CI 1.04–2.44).

## Discussion

### Linkages between caregiver sex and OVC HIV status

This study assessed how caregiver sex and other individual and household characteristics are associated with HIV infection among OVC in Tanzania. Findings revealed that OVC with male caregivers were 40% more likely to be HIV-positive than those with female caregivers. This effect remained statistically significant even after adjusting for OVC sex, caregiver HIV status, OVC nutritional status, household family size, caregiver marital status, wealth quintile, place of residence, health insurance ownership, and household co-residence. In the stratified analysis by OVC age, the association was strongest among the 0–4 years age group and declined (but with statistical significance) among 5–9 year-olds. In the older OVC age groups of 10–14 and 15–19 years, the association declined further and lost statistical significance, although the direction of the association remained.

Given the Tanzanian cultural context, when a male is the primary caregiver, likely this is because the child’s mother has died, possibly due to HIV, which increases the likelihood that the child is HIV-infected. The child may have acquired the HIV through MTCT. This has important implications for risk assessment and referral to HIV testing services (HTS) for children. As noted earlier, orphaned children living with male caregivers are likely to miss HTS because the current case-finding modalities, like index testing services, are offered to individuals who are at risk of HIV exposure from the original client, who is often the mother. Specifically, if an HTS client is a man and tests HIV-negative, the process stops [44]. This leaves a service gap for children and other family members of the male HTS client who may be at risk or already infected with HIV. Therefore, in view of this association, the current pediatric case finding strategies may be expanded by considering caregiver sex an imperative dimension for targeted HTS among OVC.

Additionally, caregiving work has traditionally been viewed as the responsibility of women and girls [54–56], especially in African communities. These traditional gender norms have been reported to exclude men and boys from becoming caregivers, thus exacerbating the caregiving burden on women [54]. This is corroborated by this study, in which about three-quarters of the OVC had female caregivers. Thus, men become caregivers not by choice, but because circumstances dictate (e.g., the child’s mother has died, and possibly there is no female relative to care for the children etc.). In this context, men are likely to provide inadequate or suboptimal care for reasons such as lack of experience in child-rearing activities. This can result into less protection of the child from HIV risks and risk-seeking behaviors. Due to traditional occupational gender norms, men generally provide more economic support to the family than women [57], but not social or direct support in the caregiving process. Although the level of parental supervision has been acknowledged as an important factor in preventing and remediating HIV in children and youth [58], evidence of men’s participation in the whole process of caregiving work is scant [59]. There is a known link between HIV and child abuse and neglect [60], whereby child abuse by male caregivers puts children at a greater risk of HIV acquisition [61]. While this study demonstrates a significant association between caregiver sex and OVC HIV infection, further research is needed to uncover and explain the causal pathways of the relationship.

### Additional risk factors for OVC HIV status

As expected, OVC with HIV-positive caregivers were more than 29 times more likely to be HIV-positive than OVC whose caregivers were HIV-negative, which is consistent with the literature [43, 62–66]. Children primarily acquire HIV from their mothers in utero, during their delivery, or while being breastfed [12]. This maybe a possible mechanism underlying the findings of this study, wherein most of the OVC in this study had female caregivers, the majority of whom were possibly their biological mothers. Therefore, although it is important to address barriers against universal coverage of HIV services to prevent MTCT, HIV risk assessment and referral to HTS and other community case-finding activities should target OVC whose caregivers are HIV-positive.

Similarly, undernourishment of any kind (moderate or severe) was significantly associated with OVC HIV-positive status, suggesting compulsory HIV testing for all undernourished children. From the literature, the relationship between nutrition and HIV is bi-directional [67, 68]: HIV compromises the immune system, leading to undernutrition, which, in turn, leads to further immune malfunction that accelerates HIV transmission and progression to AIDS [69–72]. Tanzania protocols already recommend referral of malnourished children to HTS as a case-finding approach. Because a large percentage of HIV-positive OVC were undernourished at the time of enrollment, this finding further emphasizes the importance of integrating nutrition services into community-based OVC programming and into HIV prevention and treatment programs to enhance OVC health outcomes.

There was also a residence location aspect of the variability in HIV prevalence among OVC in the study area. The study population was almost evenly split between urban and rural locations, but urban residence was associated with a 29% lower odds of HIV infection than rural residence, implying that OVC who reside in rural areas may have higher burden of HIV infection. Rates of HIV prevalence due to location were highest among OVC ages 0–4 years who resided in rural areas compared to their urban counterparts in the same age group (9.6% vs. 3.7%; see Fig. 2). The rural–urban gap in OVC HIV prevalence declined consistently with OVC age until prevalence among urban OVC exceeded that of rural OVC in the 15–19 years age category. This finding could have several interpretations. First, it could reflect cultural patterns of OVC mobility across extended family households due to vulnerability in a household, abandonment by the parent or caregiver, or death of one or more parents or caregivers [73]. Younger OVC could be more likely to stay in or be relocated to caregivers in rural areas, while older OVC could be more likely to move to urban areas for education or economic opportunities. However, there is little published data on mobility of OVC in Tanzania to substantiate these possibilities. Further, urban areas may have better exposure to HIV knowledge and awareness [74], as well as better access to health and social services [75, 76], which may have contributed to lower HIV prevalence among OVC in urban than in rural areas. The results suggest a need to target rural communities with HIV preventive, diagnostic, and treatment services as well as social welfare and case management services, especially for children under 15 years of age.

Interestingly, as mentioned before, the relationship between place of residence and OVC HIV status changed direction among the 15–19 years age group, wherein urban residence was associated with a 59% greater likelihood of HIV infection than rural residence. This finding suggests that older OVC are less likely to be long-term survivors of perinatal infection and are primarily infected through sexual transmission. While one study found greater HIV infection among 15–24 year-olds in rural than urban areas [77], many other studies around Africa have shown that HIV prevalence among adults is higher in urban than in rural areas [78–80]. This reinforces the need to address risk factors of HIV infection in orphans and vulnerable adolescents, particularly in urban areas. Further research is required to clarify the pathway by which rural or urban residence affects HIV risk among OVC.

Finally, the observed higher likelihood of OVC HIV-positive status by health insurance ownership can be associated with Pact’s previous and current support to the communities in terms of improved access to health and HIV services, whereby presence of an HIV-positive person in a household is one of the criteria to support health insurance acquisition [81].

## Conclusions

The current study demonstrates that, OVC living with male caregivers are 40% more likely to be HIV-positive than those living with female caregivers. This observation suggests that to benefit the current pediatric case finding strategies in Tanzania, considering sex of the caregiver as one of the priority factors indicating where to target case finding efforts may be worthwhile. Further qualitative and quantitative research is needed to uncover the mechanism responsible for this trend.

At the facility level, health care workers should continue to prioritize index testing for children of HIV-positive biological mothers, but the policy can expand the algorithms with HIV-positive men to identify at-risk OVC who may reside in their households. HTS providers can include questions to HIV-negative men about the known HIV status of OVC in their households. And, criteria for enrolment of OVC into social welfare programming can be reviewed from a gender lens to monitor that OVC living with male caregivers are not excluded or do not face other enrolment biases.

As such, more gender-sensitive programmatic activities targeting OVC with male caregivers are needed. For community-based programs such as the USAID Kizazi Kipya, which is built on a social welfare platform, households where the caregiver is male require additional attention during program service provision. For example, evidence–based HIV prevention approaches for orphans and vulnerable adolescents are often integrated into parenting classes that involve the adolescent and the caregiver together. Information on early infant diagnosis of HIV is integrated into early childhood development approaches. Prevention of sexual abuse is included in all parenting approaches. It is worth examining further the extent to which male caregivers are engaged in these parenting activities.

Other key dimensions that should be targeted and integrated in HIV programming efforts for improved OVC health outcomes are individual OVC characteristics such as age and nutritional status; caregiver characteristics such as HIV status and marital status; household characteristics such as health insurance status, and family size; and rural versus urban residence. These factors should be considered when setting targets for community based OVC programs engaged in community-based pediatric case-finding activities.

## Data Availability

The datasets analyzed during the current study are not publicly available due confidentiality restrictions, but are available from the corresponding author on reasonable request.

## Abbreviations

AIDS: Acquired immunodeficiency syndrome
ART: Antiretroviral therapy
CD4: Cluster of differentiation 4
CI: Confidence interval
CLHIV: Children living with HIV
FCAA: Family and Child Asset Assessment
HIV: Human immunodeficiency virus
HTS: HIV testing services
MRCC: Medical Research Coordinating Committee
MTCT: Mother-to-child transmission of HIV
MUAC: Mid-upper arm circumference
NIMR: National Institute for Medical Research
OR: Odds ratio
OVC: Orphans and vulnerable children
PCA: Principal component analysis
PLHIV: People living with HIV
PMTCT: Prevention of mother-to-child transmission of HIV
SD: Standard deviation
UNAIDS: Joint United Nations Programme on HIV/AIDS
USAID: United States Agency for International Development
